# Forage plants of an Arctic‐nesting herbivore show larger warming response in breeding than wintering grounds, potentially disrupting migration phenology

**DOI:** 10.1002/ece3.2859

**Published:** 2017-03-19

**Authors:** Thomas K. Lameris, Femke Jochems, Alexandra J. van der Graaf, Mattias Andersson, Juul Limpens, Bart A. Nolet

**Affiliations:** ^1^ Department of Animal EcologyNetherlands Institute of Ecology (NIOO)WageningenThe Netherlands; ^2^ Theoretical and Computational EcologyInstitute for Biodiversity and Ecosystem DynamicsUniversity of AmsterdamAmsterdamThe Netherlands; ^3^ Plant Ecology and Nature Conservation GroupUniversity of WageningenWageningenThe Netherlands; ^4^ GELIFES‐Groningen Institute for Evolutionary Life SciencesGroningenThe Netherlands; ^5^ Campus GotlandUppsala UniversityVisbySweden

**Keywords:** Arctic amplification, *Branta leucopsis*, migratory timing, open‐top chambers, phenological mismatch

## Abstract

During spring migration, herbivorous waterfowl breeding in the Arctic depend on peaks in the supply of nitrogen‐rich forage plants, following a “green wave” of grass growth along their flyway to fuel migration and reproduction. The effects of climate warming on forage plant growth are expected to be larger at the Arctic breeding grounds than in temperate wintering grounds, potentially disrupting this green wave and causing waterfowl to mistime their arrival on the breeding grounds. We studied the potential effect of climate warming on timing of food peaks along the migratory flyway of the Russian population of barnacle geese using a warming experiment with open‐top chambers. We measured the effect of 1.0–1.7°C experimental warming on forage plant biomass and nitrogen concentration at three sites along the migratory flyway (temperate wintering site, temperate spring stopover site, and Arctic breeding site) during 2 months for two consecutive years. We found that experimental warming increased biomass accumulation and sped up the decline in nitrogen concentration of forage plants at the Arctic breeding site but not at temperate wintering and stop‐over sites. Increasing spring temperatures in the Arctic will thus shorten the food peak of nitrogen‐rich forage at the breeding grounds. Our results further suggest an advance of the local food peak in the Arctic under 1–2°C climate warming, which will likely cause migrating geese to mistime their arrival at the breeding grounds, particularly considering the Arctic warms faster than the temperate regions. The combination of a shorter food peak and mistimed arrival is likely to decrease goose reproductive success under climate warming by reducing growth and survival of goslings after hatching.

## 1 Introduction

The matching of animal's annual cycles to peaks in food availability is considered to be an important adaptation for successful reproduction (Lack, 1968). A multitude of species match their period of reproduction to peaks in food availability in order to feed their young and to maximize their growth rates (Both & Visser, 2005). During spring migration, migratory species can also travel along a climatic gradient and match arrival on stopover sites to local peaks of food abundance along the gradient, described as the “green wave hypothesis” (Drent, Ebbinge, & Weijand, 1978; Shariatinajafabadi et al., 2014; Thorup et al., 2017; van der Graaf, Stahl, Klimkowska, Bakker, & Drent, 2006). This strategy is especially important for species which partly rely on capital body stores accumulated at staging sites for egg formation and incubation, such as geese (Drent et al., 2007; Gauthier, Bêty, & Hobson, 2003; Hahn, Loonen, & Klaassen, 2011). The matching of migration timing to peaks in food availability could be strongly disrupted by global climate warming when food peaks change asynchronously over the migratory flyway (Klaassen, Hoye, Nolet, & Buttemer, 2012).

Global climate warming has advanced the phenology of spring events, such as the leafing and flowering of trees and emergence of insects (Menzel et al., 2006; Parmesan & Yohe, 2003; Visser & Both, 2005). Several bird species have been able to advance their laying date accordingly (Visser & Both, 2005), while others, notably long‐distance migrants, have not (Both & Visser, 2001; Clausen & Clausen, 2013; Møller, Rubolini, & Lehikoinen, 2008). Such a mismatch in intertrophic relationships can have large consequences for reproductive success and, ultimately, population size (Both & Visser, 2001; Miller‐Rushing, Hoye, Inouye, & Post, 2010; Møller et al., 2008; van Gils et al., 2016). Migratory species are more vulnerable to these mismatches, as changes in climate are often not correlated between their wintering sites and breeding grounds (Emmenegger et al., 2016; Kölzsch et al., 2015). In the Arctic region, climate warming is expected to be more severe than the global average, a process called arctic amplification (Serreze, Barrett, Stroeve, Kindig, & Holland, 2009; Stocker et al., 2013), and rapid advancement of the onset of spring inducing strong phenological responses of plants and animals are already found in the Arctic (Høye, Post, Meltofte, Schmidt, & Forchhammer, 2007; Post et al., 2009). Accelerated warming in the Arctic is expected to cause food peaks in Arctic regions to advance at a faster rate than in temperate regions, which could cause mismatches especially for Arctic long‐distance migrants (McKinnon, Picotin, Bolduc, Juillet, & Bêty, 2012).

Arctic long‐distance migrants, such as geese, take benefit from both temperate and Arctic food peaks to maximize reproductive success, using the food peaks in temperate regions to fuel migratory flight, egg production and incubation (Drent et al., 2007), and the food peak in the Arctic to rear their chicks (Doiron, Gauthier, & Lévesque, 2015; van der Graaf et al., 2006). As food peaks along the migratory flyway match the onset of spring (van der Graaf et al., 2006; van Eerden, Drent, Stahl, & Bakker, 2005), geese use these to time their migration (Duriez et al., 2009; Shariatinajafabadi et al., 2014; van Wijk et al., 2012). Food peak phenology might be differentially affected by climate warming at different latitudes, as plants in colder, higher latitudes might be more responsive to temperature increase (Havström, Callaghan, & Jonasson, 1993). When food peaks along the flyway advance at different rates, this can affect migration and reproduction of Arctic nesting geese in two ways: (1) When food peaks in the Arctic advance faster than in temperate regions, the period between the food peaks in temperate zones and the food peak on the breeding grounds becomes shorter. This may make it more difficult for the geese to benefit from multiple food peaks along a green wave (van der Graaf et al., 2006), causing them to arrive on the breeding grounds either later or with less body stores to initiate breeding. (2) When food peaks in the Arctic and in temperate regions advance at different rates, the cues which the geese currently use to time their departure might prove to be no longer valid (Emmenegger et al., 2016; McNamara, Barta, Klaassen, & Bauer, 2011). Geese will then suffer from reduced capacity to predict an earlier peak at the Arctic site and arrive too late (Kölzsch et al., 2015).

Already now, some Arctic nesting geese have been found to initiate nesting too late under increased spring temperatures in the Arctic, resulting in a mismatch between hatching date and high‐quality food availability (Dickey, Gauthier, & Cadieux, 2008), reducing gosling growth rates (Doiron et al., 2015) and possibly driving declines in reproductive success (Clausen & Clausen, 2013). As Arctic food peaks are predicted to advance under increasing temperatures (Doiron, Gauthier, & Lévesque, 2014), these mismatches will likely become stronger under amplified climate warming in the Arctic (Doiron et al., 2014, 2015). However, as food peak phenology might be differentially affected at different latitudes, it is currently unclear how an advancement of food peaks in the Arctic relates to advancement of food peaks along the migratory flyway, and thus whether it can lead to a mismatch of migratory timing. In order to make predictions on the extent of such a mismatch, it is necessary to study how food peaks along the complete migratory flyway will advance under predicted climate change.

We studied the potential effect of climate warming on the advancement of food peaks along the migratory flyway of an Arctic nesting goose. We examined the impact of experimental warming on forage plant biomass, nitrogen concentration, and peak nitrogen availability, using open‐top chambers at a wintering, staging, and breeding site. We then applied the empirically determined relationship between growing degree days, that is, the sum of mean daily temperatures above a certain temperature threshold (van Wijk et al., 2012), and nitrogen/nitrogen concentration to calculate the potential advancement of food peaks under 1–2°C climate warming. We specifically test the hypothesis that climate warming advances food peaks more in the Arctic breeding site than in temperate wintering and stopover sites, thus shortening the period between subsequent food peaks along the migratory flyway.

## 2 Methods

### 2.1 Study system and study sites

**Figure 1:**
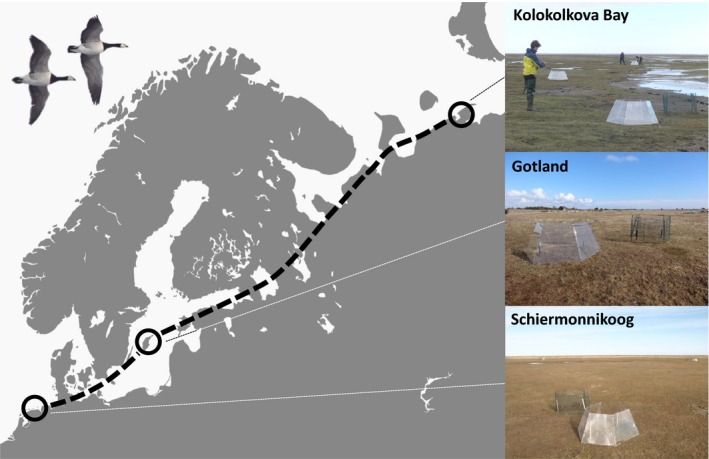
Our study sites (black circles) along the spring migration route of the Russian barnacle goose population (black‐dashed line). Dotted lines connect to photographs of the study sites showing the experimental setup, with the experiment plots covered by open‐top chambers and fenced control plots. Photographs were taken in April 2015 (Schiermonnikoog, photograph by TKL and Gotland, photograph by FJ) and June 2015 (Kolokolkova Bay, photograph by BAN)

As a study system, we used the migratory flyway of the Russian population of barnacle geese *Branta leucopsis*, which stretches between their wintering areas in northwestern Europe, along the Baltic Sea and White Sea to the breeding grounds along the Barents Sea coast in Northern Russia. This is a well‐known model system to study bird migratory timing in relation to the green wave (Shariatinajafabadi et al., 2014; van der Graaf et al., 2006) and climatic variables (Kölzsch et al., 2015). Our study sites are located in preferred feeding salt marsh habitats at three sites along this migratory flyway: one temperate wintering site, one temperate spring stopover site, and one Arctic breeding site (Figure 1). The first temperate site is situated on the island of Schiermonnikoog in the Wadden Sea, the Netherlands, which is both a wintering and spring staging site for barnacle geese (53°30′N, 6°10′E). The second temperate site is located on the island of Gotland in the Baltic Sea (57°07′N, 18°27′E), a traditional stopover site for migrating barnacle geese in April and May (van der Graaf, Stahl, Veeneklaas, & Bakker, 2007). The Arctic site is at the Kolokolkova Bay on the Barents Sea coast, northern Russia (68°35′N, 52°20′E), which hosts a breeding colony of barnacle geese (van der Jeugd et al., 2003). Barnacle geese have been breeding in this area since at least 1994 (Syroechkovsky Jr, 1995, and the colony now (2015) comprises approximately 600 breeding pairs (T.L. unpublished data).

### 2.2 Experimental setup

We conducted a warming experiment at our study sites in the spring of 2014 and 2015 to study the effect of warming on forage plants for barnacle geese. At temperate sites, we specifically studied Red Fescue (*Festuca rubra*), and at the Arctic site, we studied Hoppner's Sedge (*Carex subspathacea*). The use of different plant species on different sites could pose a confounding factor, as differences between sites could alternatively be explained by the differences between species. However, by studying the main forage plants for barnacle geese for these specific sites (van der Graaf, 2006; van der Graaf, Lavrinenko, Elsakov, Van Eerden, & Stahl, 2004; van der Graaf et al., 2006), we are able to study the effect of warming in the context of goose migration timing rather than to gain a specific understanding of the effects of warming on vegetation at different latitudes. We experimentally warmed vegetation plots at small scale using hexagonal open‐top chambers (OTCs) with a basal diameter of 100 cm, a height of 50 cm, and a side angle of 60°, made from LEXAN polycarbonate (non‐UV resistant; Figure 1). Our open‐top chambers were constructed according to the protocol for the International Tundra Experiment (ITEX) program, which are used in many studies of climate warming (Bokhorst et al., 2013; Elmendorf et al., 2012; Marion et al., 1997; Molau & Edlund, 1996). Open‐top chambers typically warm the soil temperature with 1–3°C (Marion et al., 1997; Molau & Edlund, 1996), which is line with expected climate warming in this century (Stocker et al., 2013). For every warmed plot, we placed a control plot at 1 m distance, which was fenced with chicken wire (1 cm mesh size, 50 cm in height) to prevent geese and other herbivores from entering the plot (Figure 1). Five open‐top chambers and five control plots were placed with at least 50 m distance on each study site for two months during the growing season when the geese were preparing for migration (March–April; Schiermonnikoog), staging on a stopover site during migration (April–May; Gotland) or nesting/rearing offspring on the breeding grounds (early/mid‐June–early/mid‐August; Kolokolkova Bay, see Table S1 for exact dates). Plots were placed in low–middle saltmarsh where forage plant abundance exceeded 50% cover (van der Graaf et al., 2004, 2007; van Wijnen, Bakker, & de Vries, 1997). In 2015, plots were placed at least 50 m from locations used in 2014 to avoid repeated measuring on the same plot. In the Kolokolkova Bay, the experiment was set up after disappearance of sea ice from the saltmarsh, which was 10 days earlier in 2015 compared to 2014. This could have caused differences between years in the amount of warmed days to which the experimental plots were exposed, which in turn could affect measured parameters. We expect that the general pattern between sites would not be affected.

### 2.3 Data collection

#### 2.3.1 Vegetation

Every 14 days, we (1) counted the density of living green tillers of the forage plants (individual sprouts consisting of 1–3 leaves) and (2) collected individual tillers using pairs of tweezers, both in randomly placed 5 × 5 cm surface squares (2014: 10 squares, 3 tillers collected per square; 2015: 5 squares, 5 tillers collected per square). Measurements were almost always conducted on a single day, and otherwise on (up to three) consecutive days. To reduce the time it took to conduct the measurements, we adjusted the measurement protocol in 2015 to count less squares. We tested that when counting 5 squares, all counts' values would fall within the confidence intervals of the original counts using 10 squares (Appendix S1, Fig. S1). We simultaneously increased the number of collected tillers per square in order to collect enough biomass for determining nitrogen concentrations. Once a square was used for data collection, it was excluded for the remainder of the experiment. After collection, tillers were dried at room temperature and thereafter stored in paper bags for 1–2 months. Samples were re‐examined in the laboratory to remove soil particles and dead material, after which they were oven‐dried at 60°C for 48 h and weighed to the nearest milligram. Samples were then grinded to 1 mm particles using a bead mill with steel beads (QIAGEN TissueLyser II), after which nitrogen (N) and carbon (C) content (% of dry weight) were determined on 3–5 mg powdered material in 6‐mm‐diameter metal cups, using a C:N analyser (Flash EA 1112 analyzer from Thermo Fisher Scientific Inc. Waltham, USA). We determined total aboveground biomass (dry weight in g m^−2^) by multiplying the average tiller weight with average tiller density count per square, multiplying by 400 as we measured in 5 × 5 cm squares. We combined the measures of aboveground biomass and N concentration to calculate total aboveground nitrogen (in g N m^−2^). We placed temperature loggers (Ibutton Thermochron 1922L) in the center of each plot, 2 cm below the surface, which measured soil temperature every 24 min at 0.1°C accuracy. For every site, the loggers recorded temperature from the day of the first measurements until the day of the last measurement.

#### 2.3.2 Growing degree days

We calculated growing degree days for every year, study site, and plot using a combination of the temperature data collected in our plots and temperature data from nearby weather stations. We used the daily mean air temperature data from 2014 and 2015 of the weather station located closest to each study site (Lauwersoog; 8.2 km from Schiermonnikoog, Visby: 58.8 km from Gotland site, Naryan‐Mar/Konstantinovsky: 121.8/107.1 km from Kolokolkova Bay site; more information in Appendix S1). We acquired these temperature data from national weather services and from the Russian weather site “RP5” (KNMI; Swedish Meteorological and Hydrological Institute; www.rp5.ru). To acquire mean daily temperature specifically for each study site and plot for the entire years 2014 and 2015, we used mean daily temperature data from our plots and added data from weather stations for the months outside the experiment. For the complete dataset, we calculated growing degree days (GDD) according to van Wijk et al. (2012), using 0°C as a threshold temperature for grass growth (Gallagher, 1979).

### 2.4 Statistics

We calculated the daily mean and maximum temperature per plot, treatment, and site and tested the effect of our warming treatment by running linear‐mixed models using the package “*lme4*” in R 3.0.2 (R Core Team, 2014). We fitted plot as a random factor and included fixed factors treatment, site, year, and the interactions between treatment and year and treatment and site. We fitted treatment, year, and site as fixed factors and plot (nested in site and year) as a random factor.

We tested the effect of warming on aboveground biomass (g m^2^), nitrogen concentration (%), and aboveground nitrogen (g m^2^) by running linear‐mixed models. We fitted plot (nested in site and year) as random factor in our models and included multiple fixed effects, including days (since start of the experiment), the quadratic term of days, year, site, warming treatment, the interaction between warming treatment and days, and an interaction effect between days and site. In models including the fixed factor of treatment, we tested whether the treatment led to an increase or decrease in biomass or nitrogen, while in models including the interaction between treatment and days and the quadratic term of days, we tested whether the treatment advanced or delayed the peak value of biomass or nitrogen. Models with combinations of variables were compared using Akaike's information criterion (AIC_c_; Burnham & Anderson, 2004) and we chose the model with the lowest AIC_c_ value as our final model. We tested for significance of fixed factors by comparing the final model with a reduced model in which the fixed factor was absent, using a likelihood ratio test. As site and the interaction effect of days and site were significant in most models, we tested models separately per site.

### 2.5 Food peak advancement

To investigate the effect of warming on the advancement of food peaks, we additionally conducted an analysis in which we using growing degree days to predict the moment of the food peak under climate warming. Local climate and plant phenology are closely linked (Cleland, Chuine, Menzel, Mooney, & Schwartz, 2007; van der Graaf et al., 2006) and peaks in food quality can be predicted by GDD (Botta, Viovy, & Ciais, 2000; Si, Xin, Prins, de Boer, & Gong, 2015). As we advanced the GDD under our warming treatment, we expected food peaks to advance, but only if our treatment affected plant growth. We defined the food peak as the peak in (aboveground) nitrogen (g m^2^) at temperate sites, while for the Arctic site, we defined the food peak as the peak in nitrogen concentration (%), as food requirements differ between adults and chicks and thus between sites. Food peaks at temperate sites are mostly determined by the combination of high nitrogen concentration and aboveground biomass (van der Graaf et al., 2006), facilitating adult geese to rapidly accumulate fat reserves prior to and during migration. The food peak to which geese should time hatching of their eggs in the Arctic is on the other hand determined by the food requirements of the goslings, which should forage on short vegetation, which is high in nitrogen concentration in order to grow fast (Doiron et al., 2015; Richman, Leafloor, Karasov, & Mcwilliams, 2015).

**Figure 2:**
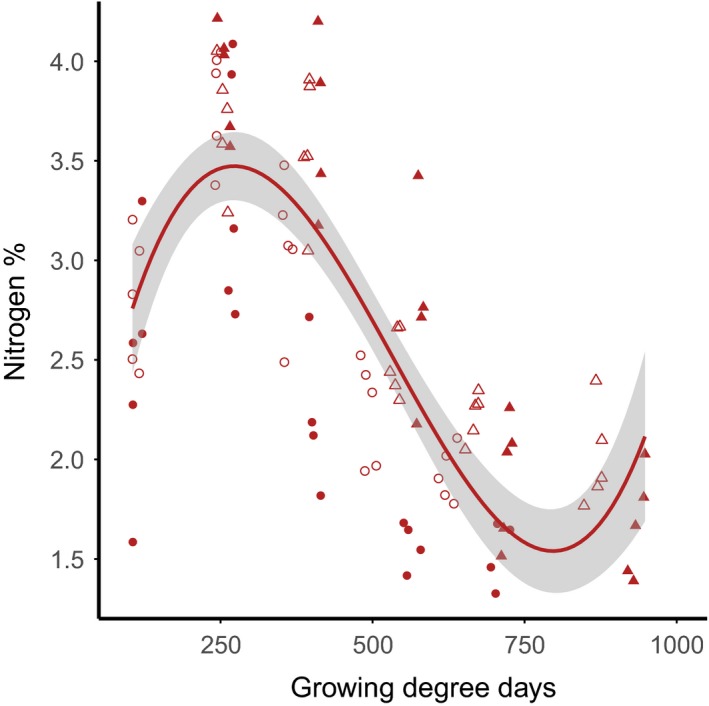
Nitrogen concentration (%) over growing degree days (GDD) for the Arctic site Kolokolkova Bay. Open symbols represent measurements from the control treatment, closed symbols from the warmed treatment. Circles represent data from 2014, triangles data from 2015. The line shows the polynomial regression, with the shaded area representing the 95% CI of the linear functions. Specific regression function: NC_k_ = 1.364 + 1.748*e* ^−02^. GDD_d_ + −4.305*e* ^−05^. ${\text{GDD}}_{d}^{2}$ + 2.684*e* ^−08^. ${\text{GDD}}_{d}^{3}$ (adjusted *R* ^2^ = 0.619)

As our treatment only affects plant growth at the Arctic site (see results), we assumed the timing of the food peaks at our temperate sites to be unaffected by climate warming. We calculated the timing of the Arctic food peak by applying the empirically determined polynomial regression between nitrogen concentration (%; NC) and growing degree days (GDD) under climate warming. We determined these relations by running linear‐mixed models on our experimental data. In our models, GDD explained nitrogen concentration better (i.e., lower AIC_c_) than days since start of the experiment or Julian days. This relationship did not differ between warmed and control treatments, but differed significantly between years (χ^2^ = 40.26, *p* < .001), as we started measuring before nitrogen concentration peaked in 2014 but not in 2015. We included both years in the analysis, as the peak in nitrogen concentration was reached at approximately the same GDD in both years (Figure 2). We thus obtained the intercept and the fixed‐effects regression coefficients (a, b, and c) from linear‐mixed models including both treatments and both years. This allowed us to create a growing degree day model to calculate nitrogen concentration (NC) for any given Julian day (d) using the GDD of the specific site and day:

(1) $${\text{NC}}_{d}=\text{intercept}+a{\left({\text{GDD}}_{d}\right)}^{3}+b{\left({\text{GDD}}_{d}\right)}^{2}+c\left({\text{GDD}}_{d}\right)$$

We validated the growing degree day model for nitrogen concentration with data collected in the field (Appendix S1, Fig. S2). We applied the empirically determined relationships to calculate increase in nitrogen concentration under 0.0–2.0°C warming, as is this is consistent with the rate of warming in our warming treatment. To calculate the GDD under warming, we first acquired the baseline temperatures from the average temperature per Julian day over the period 2005–2015, specific per site (data from weather stations described above). We then added +1, +1.5, and + 2.0°C to the baseline temperatures. From these temperatures, we calculated GDD as described above and then used these GDD values in formula one to calculate nitrogen concentration over Julian days. For every temperature increase, we then determined the Julian day at which nitrogen concentration reached its maximum value, which is the “peak.” We then calculated the advancement of the food peak respective to a the food peak without warming.

## 3 Results

### 3.1 Temperature in open‐top chambers

The open‐top chambers increased the soil temperature in plots on average by 1.0–1.7°C for all sites. Both mean and maximum daily temperature were significantly higher in plots warmed by open‐top chambers than in control plots (mean temperature: χ^2^ = 182.76, *df* = 1, *p *< .001; maximum temperature: χ^2^ = 178.08, *df* = 1, *p* < .001). The interaction effect of treatment and year was included in the best model for maximum daily temperature, but this was only marginally significant (χ^2^ = 2.72, *df* = 1, *p *= .099). There was no difference in the treatment effect between sites (Appendix S1, Tables S2 and S3).

### 3.2 Warming effects

**Figure 3:**
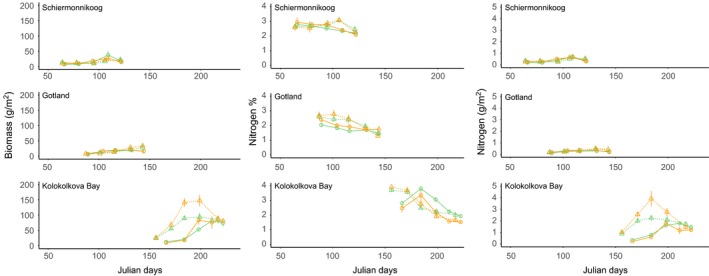
Change in biomass (g m^‐2^), nitrogen concentration (%), and nitrogen (g m^‐2^) over time (in Julian days) of forage plants for both control (green lines) and warmed (orange lines) treatments, in 2014 (circles and solid lines) and 2015 (triangles and dotted lines)

The effect of warming on plant growth differed between sites and only at the Arctic breeding site Kolokolkova Bay did experimental warming affect forage plant growth and development (Figure 3, Table 1). Here, the warming treatment was included in the best model for aboveground biomass and nitrogen concentration (Appendix S1, Table S4), in which it was a significant predictor (GLMM: aboveground biomass χ^2 ^= 7.588, *p* = .006; nitrogen concentration χ^2^ = 6.300, *p* = .012). In warmed plots, the increase in biomass was significantly higher while the decline in nitrogen concentration was significantly faster. The interaction of the warming treatment and days since start of the experiment was not significant. At the Gotland and Schiermonnikoog sites, the treatment effect nor the interaction effect was ever significant (Table 1).

**Table 1:** Significant fixed factors with unstandardized coefficients in GLMMs run for biomass, nitrogen concentration, and aboveground nitrogen (g m^2^)

Test variable	Site	Days	Days^2^	Treatment	Year	Days × Treatment	Site	Site × Treatment
Biomass (g m^−2^)	All	0.967***	−0.008**	1.737**	10.408**		44.829***	12.558*
	Schiermonnikoog	0.367*						
	Gotland	0.416**						
	Kolokolkova Bay	3.750***	−0.039***	14.521**	34.800***			
Nitrogen concentration (%)	All	0.009*	−0.0004***	0.092*	0.299***		0.954***	−0.353*
	Schiermonnikoog	*0.012*	−0.0003**		*0.144*			
	Gotland		−0.0003***	*0.199*	0.362*			
	Kolokolkova Bay		−0.0005***	−0.268*	0.279**			
Nitrogen (g m^−2^)	All	0.029***	−0.0004***		0.306**		1.246***	
	Schiermonnikoog	0.012*						
	Gotland	0.010**						
	Kolokolkova Bay	0.095***	−0.001***		1.025***			

Asterisks denote significant effects (*p* < .05: *; *p* < .01: **; *p* < .001: ***), and italic values denote marginally significant effects. Models for which site is indicated in the second column were run separately for a single site. The variables Site and Site × treatment were only included in models which were run for all sites.

### 3.3 Food peak advancement

As our warming treatment did not affect plant growth in our experiment at temperate sites, we assume that timing of temperate food peaks is not affected by the degree of climate warming expected in the coming century. At the Arctic site, our growing degree day analysis predicted the food peak in nitrogen concentration to advance by 4, 5, and 7 days under +1.0, +1.5, and +2.0°C warming, respectively.

## 4 Discussion

We found that experimentally increasing temperatures had a strong effect on forage plants of barnacle geese at our Arctic site but not at the two temperate sites. At the Arctic site, warming resulted in an increase in the peak of biomass and faster decline of nitrogen concentration, whereas warming had no such effects at the temperate sites. Under a 1–2°C climate warming, food peaks are unlikely to advance at temperate sites, while food peaks at the Arctic site are predicted to advance up to 7 days.

### 4.1 Effects of experimental warming in the Arctic

The increase in 1.0–1.7°C in our warmed treatment did not differ between sites and years and was in line with other studies using ITEX open‐top chambers in similar climatic regions (Doiron et al., 2014; Marion et al., 1997; Rustad et al., 2001). In our Arctic site, aboveground biomass accumulated faster and to a higher peak level in warmed plots, while nitrogen concentration in the shoots was lower. This is consistent with other experimental warming studies on graminoids in the Arctic region (Doiron et al., 2014; Jónsdóttir, Khitun, & Stenström, 2005). Plants in Arctic regions have in general been found to be more responsive to increased temperatures in summer (Havström et al., 1993; van der Wal & Stien, 2014). Warming, either experimental or natural, will have proportionally larger effects in Arctic regions compared to temperate regions due to colder average temperatures and can result in prolongation of the growing season (van der Wal & Stien, 2014), which is otherwise inhibited by low summer temperatures (Atkin, Bruhn, Hurry, & Tjoelker, 1999). A seasonal decline of nitrogen concentration occurs simultaneously with increasing aboveground biomass, a pattern generally found in Arctic plants as they age (Chapin, Cleve, & Tieszen, 1975; Lepage, Gauthier, & Reed, 1998; van der Graaf et al., 2006). The accelerated decline of nitrogen under experimental warming could be a dilution effect which occurs during a simultaneous increase in carbon‐rich plant tissues as plant productivity is increased (Day, Ruhland, & Xiong, 2008; Doiron et al., 2014; Tolvanen & Henry, 2001), although we do not find carbon concentration to be increased in warmed plots. Finally, as previously suggested by Doiron et al. (2014), warming appeared to increase aboveground nitrogen and thus the height of the food peak, although this was not significant. The amplified effect of warming at Arctic sites is in line with results from other studies: meta‐analyses report greater positive effects of warming on plant productivity in colder regions (Elmendorf et al., 2012; Rustad et al., 2001).

### 4.2 Food peak advancement

The interaction of days since start of the experiment and the warming treatment was never significant for any of our sites, suggesting that the warming treatment did not advance the moment peak food availability, either in nitrogen (g m^‐2^) or in nitrogen concentration. The detection of small advancements in timing of the food peak under experimental warming might, however, be weakened by differences in the height of the food peak between years and treatments and the low frequency of our measurements (i.e., once every 14 days). When we use our growing degree day model, we predict food peaks to advance 7 days in the Arctic under a 2°C climate warming. Contrastingly, food peaks are unlikely to advance at temperate sites under warming up to 1.7°C, as out warming treatment did not affect plant growth. The larger warming response of Arctic forage plants which we find can more strongly advance peaks in food availability further along the migratory flyway and thus give rise to mismatches between bird migration and peak food availability (Doiron et al., 2015; Kölzsch et al., 2015; Meltofte et al., 2007).

### 4.3 Disruption of the green wave

We find that a temperature increase in 1.0 to 1.7°C had an larger effect of forage plants growth in the Arctic, leading to a stronger increase in biomass and a stronger decline of nitrogen concentration in plants. This can be problematic for small goslings, which cannot access tall grass swards and need short, nitrogen‐rich grass for rapid growth after hatching (Doiron et al., 2014; Richman et al., 2015). In the breeding grounds of barnacle geese, climate warming is expected to result in a shorter “food peak” during which this high‐quality forage is available. When goslings feed on lower quality forage after the food peak, they suffer from reduced growth (Doiron et al., 2015; Lepage et al. 2005) and a shorter food peak could thus strongly reduce gosling growth and survival, as has been found for Arctic‐nesting Sanderlings (Reneerkens et al., 2016). At temperate wintering and staging sites, experimental warming did not affect forage quality, and fat deposition rates of adult geese preparing for spring migration is thus unlikely to change under climate warming. If the departure date from staging sites is triggered by a seasonal decline in fat deposition rate (Prop, Black, & Shimmings, 2003), geese would not advance their migration timing in temperate regions under climate warming.

In addition, under a 2°C, we predict an advancement of the food peak at the Arctic site but not at temperate sites. A similar climate warming might thus shorten the period between food peaks in the temperate wintering area and the Arctic breeding area. Under a shortening of this period, geese might not have the time to both exploit temperate food peaks prior to migration and still arrive on the breeding grounds on time (Meltofte et al., 2007). Also, as geese time their spring migration according to peaks of nitrogen (van der Graaf et al., 2006), the lack of advancement of food peaks in temperate regions could deteriorate the ability of the geese to predict food conditions on the Arctic breeding grounds (Kölzsch et al., 2015). Either one or the combination of these effects is likely to result in a mismatch between goose migration phenology and peak food availability (Dickey et al., 2008), which has been shown to strongly reduce gosling growth (Doiron et al., 2015). In combination with a shortened food peak in the Arctic, the negative effects on gosling growth will be amplified.

Under Arctic amplification, the temperature rise in the Arctic is predicted to be 2.2 to 2.4 times higher than the global average (Serreze et al., 2009; Stocker et al., 2013). In addition, high inter‐annual variability of the Arctic climate can cause extreme early springs in some years (Gauthier et al., 2013), during which a shortening of the Arctic food peak is likely to occur. Under amplified Arctic warming, the period between food peaks will be shortened even more, increasing the chance of mismatched migration phenology of geese.

## 5 Conclusions

To study the effects of climate warming on migratory organisms, spring phenology of their breeding areas has to be seen in connection with their wintering and staging areas along the migratory flyway (Emmenegger et al., 2016). From this viewpoint, we show that climate warming can have a strong deteriorating effect on forage quality in the Arctic breeding grounds, potentially reducing gosling growth, but will not affect forage quality on temperate wintering grounds for staging adult geese. In addition, an advancement of the food peak in the Arctic but not at temperate sites can disrupt the timing between food peaks along the migratory flyway in our study system, which can cause goose migration phenology to become mismatched, particularly considering the Arctic warms faster than the temperate regions (Stocker et al., 2013). The combined effect of a mismatched food peak which becomes shorter as the climate warms will likely have strong impacts on goose reproductive success under climate warming by reducing growth and survival of goslings after hatching.

## Conflict of Interest

None declared.
