# Supplementary material for: Forage plants of an Arctic‐nesting herbivore show larger warming response in breeding than wintering grounds, potentially disrupting migration phenology
Source: Ecol Evol. 2017 Mar 19;7(8):2652–60. doi: 10.1002/ece3.2859 (PMC5395431; doi:10.1002/ece3.2859)
Supplement: Supplementary file 1 [file ECE3-7-2652-s001.docx]

Supplementary material Appendix S1 to:

Lameris, T.K. et al. Forage plants of an Arctic-nesting herbivore show larger warming response in breeding than wintering grounds, potentially disrupting migration phenology. Ecology & Evolution 000: 000-000


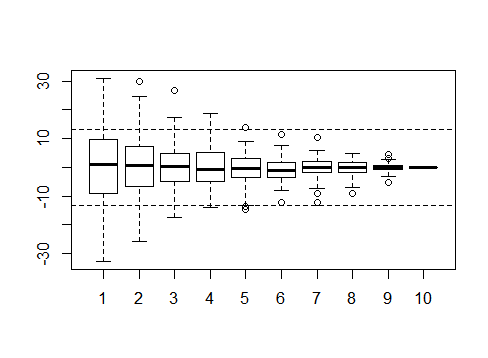


**Figure S1:** Boxplots of the average count values when using 1 to 10 squares for counting. Horizontal dotted lines delineate the 95% confidence intervals of the counts when using 10 squares.

**Table S1:** All measurement moments during the experiment for all sites and both years 2014 and 2015. The experimental set-up was put up at the day of the first measurement (moment 1) and taken down after the last measurement (moment 5).

| **Measurement moment** | **Site & year** | | | | | |
| --- | --- | --- | --- | --- | --- | --- |
|  | ***Schiermonnikoog*** | | ***Gotland*** | | ***Kolokolkova Bay*** | |
|  | *2014* | *2015* | *2014* | *2015* | *2014* | *2015* |
| 1 | 7 March | 5 March | 30 March | 28 March | 15 June | 5 June |
| 2 | 21 March | 19 March | 12 April | 11 April | 3 July | 20 June |
| 3 | 4 April | 5 April | 26 April | 25 April | 17 July | 3 July |
| 4 | 19 April | 16 April | 10 May | 11 May | 30 July | 18 July |
| 5 | 2 May | 1 May | 24 May | 23 May | 10 August | 5 August |

**Temperature data**

We acquired temperature data from weather stations located closest to our study sites. For the Schiermonnikoog site this was the weather station of Lauwersoog (data from Royal Netherlands Meteorological Institute; www.knmi.nl), located 8.2 km from our study site. For the Gotland site this was Visby, Gotland (data from Swedish Meteorological and Hydrological Institute; www.smhi.nl ) located 58.8 km away from our study site (Grotlingbo-udd). For the Kolokolkova Bay site we gathered temperature data from two weather stations (data from Raspisaniye Pogodi Ltd: [www.rp5.ru](http://www.rp5.ru) and the all-Russian scientific institute for hydro-meteorological information: www.meteo.ru), as these were either located far away from the study site (Cape Konstantinovsky, 121.8 km) or further away from the coast than our study site (Naryan-Mar, 107.1 km) which could be of influence for the temperature. For all sites we checked whether daily average temperature measured by the weather station matched the daily temperature measured by our Ibuttons at the study site during the period of study. At the Kolokolkova Bay site differences were often large (>2˚C) and we used the average of both weather stations to create a closer match.

**Validation of growing degree day model**

We validated our growing degree day model on nitrogen concentration for our Arctic study site using data gathered by SvdG on nitrogen concentration in *Carex subspathacea* in 2003 at out Arctic study site in the Pechora delta (van der Graaf et al. 2006). We used temperature data as collected on the study site between 7^th^ of June and 1^st^ of August, which we complemented with temperature data from weather stations as described above. From these temperature data we calculated growing degree days and used our model to calculate nitrogen concentration for every Julian day. We compared the moment of the nitrogen concentration food peak as predicted by our model and as measured at our study site. The model predicted the food peak to occur at day 188 (7^th^ of July), while the highest nitrogen concentration was measured at day 184 (3th of July; figure S2)

**Figure S2:** Comparison of our growing degree day model in nitrogen concentration at the Arctic site (red line) for temperature data from 2003, compared with actual data on nitrogen concentration in *Carex subspathacea* (blue diamonds).

**Table S2:** Mean, maximum and minimum daily differences in Celsius degrees between warmed and control treatments, separated for year and location.

|  | 2014 | | | 2015 | | |
| --- | --- | --- | --- | --- | --- | --- |
|  | min | mean | max | min | mean | max |
| Schiermonnikoog | 0.87 ± 0.45 | 1.04 ± 0.14 | 1.30 ± 0.56 | 1.13 ± 0.70 | 1.32 ± 0.21 | 1.90 ± 0.97 |
| Gotland | 1.10 ± 0.45 | 1.68 ± 0.51 | 2.90 ± 1.26 | 0.60 ± 0.36 | 1.37 ± 0.27 | 2.00 ± 0.84 |
| Kolokolkova Bay | 0.87 ± 0.51 | 1.29 ± 0.33 | 1.80 ± 0.72 | 0.77 ± 0.58 | 0.99 ± 0.19 | 1.50 ± 1.19 |

**Table S3:** Final GLMMs for mean and maximum temperature with coefficient values for fixed effects. Asterisks denote significant effects (p < 0.05: *; p < 0.01: **; p < 0.001: ***), italic values denote marginally significant effects.

| Test variable | Intercept | Site | Treatment | Year | Treatment*  Site | Treatment*  Year | degrees of  freedom | Log  likelihood | AICc |
| --- | --- | --- | --- | --- | --- | --- | --- | --- | --- |
| Mean temperature | 9.837 | -0.78** | 1.28*** |  |  |  | 7 | -10602.56 | 21217.1 |
| Maximum temperature | 1330.00 | -1.26*** | 865.37*** | -0.59*** |  | *-0.43* | 8 | -11957.84 | 23931.7 |

**Table S4:** Final GLMMs for biomass, nitrogen content and nutrient biomass with coefficient values for fixed effects.

|  | Intercept | Days since start | Days ^2 | Treatment | Year | Site | Site × treatment | degrees of freedom | Log likelihood | AIC_c_ |
| --- | --- | --- | --- | --- | --- | --- | --- | --- | --- | --- |
| **Biomass** |  |  |  |  |  |  |  |  |  |  |
| *All sites* | -8.729 | 0.967 | -0.008 | 1.737 | 10.408 | 44.829 | 12.558 | 11 | -1733.56 | 3489.8 |
| *Kolokolkova Bay* | -21.3 | 3.750 | -0.039 | 14.521 | 34.800 |  |  | 7 | -451.48 | 918.2 |
| *Gotland* | 5.749 | 0.416 | -0.002 | 1.417 |  |  |  | 6 | -341.03 | 695.0 |
| *Schiermonnikoog* | 9.049 | 0.367 | -0.002 |  | -1.803 |  |  | 6 | 355.28 | 723.5 |
| **Nitrogen concentration (%)** |  |  |  |  |  |  |  |  |  |  |
| *All sites* | 1.936 | 0.009 | -0.0004 | 0.092 | 0.299 | 0.954 | -0.353 | 11 | -275.17 | 573.1 |
| *Kolokolkova Bay* | 3.314 |  | -0.0005 | -0.268 | 0.279 |  |  | 6 | -71.14 | 155.2 |
| *Gotland* | 2.122 |  | -0.0003 | 0.199 | 0.362 |  |  | 6 | -433.01 | 78.9 |
| *Schiermonnikoog* | 2.597 | 0.012 | -0.0003 |  | 0.144 |  |  | 6 | 43.50 | 100.0 |
| **Nitrogen (g/m^2^)** |  |  |  |  |  |  |  |  |  |  |
| *All sites* | -0.235 | 0.029 | -0.0004 |  | 0.306 | 1.246 |  | 8 | -224.04 | 464.5 |
| *Kolokolkova Bay* | -0.149 | 0.095 | -0.001 |  | 1.025 |  |  | 6 | -86.50 | 186.0 |
| *Gotland* | 0.126 | 0.010 | -0.0001 |  | 0.055 |  |  | 6 | 63.48 | 114.0 |
| *Schiermonnikoog* | 0.220 | 0.012 | -0.0001 |  | 0.010 |  |  | 6 | 15.06 | -17.1 |
